# Synergistic effects of crop straw formulations on *Stropharia rugosoannulata* cultivation and soil microecology in continuous cropping systems

**DOI:** 10.1128/spectrum.02582-25

**Published:** 2026-05-26

**Authors:** Mengjiao Ding, Nianjie Shang, Lukuo Zhou, Hao Shu, Dengxiang Liu, Taibo Liang, Yunfei Ma, Weichang Gao, Yanling Zhang

**Affiliations:** 1 Zhengzhou Tobacco Research Institute of CNTC 154489 , Zhengzhou, China; 2 College of Tobacco Science of Guizhou University 71206, Guiyang, China; 3 Guizhou Provincial Key Laboratory for Tobacco Quality, College of Tobacco Science, Guizhou University 71206, Guiyang, China; 4 Institute of Crop Germplasm Resources, Guizhou Academy of Agricultural Sciences 74721, Guiyang, China; 5 Hunan Tobacco Company Chenzhou City Company, Chenzhou, People's Republic of China; 6 Hubei Xinye Tobacco Slice Development Co., Ltd, Wuhan, People's Republic of China; 7 Guizhou Tobacco Company Bijie Region Tobacco Company, Bijie, China; 8 Guizhou Academy of Tobacco Science 499147 , Guiyang, Guizhou, China; The Ohio State University, Columbus, Ohio, USA

**Keywords:** continuous‐cropping system, environmental factors, microbial community, nutrient cycling

## Abstract

Biological amendment strategies effectively enhance the physical and chemical properties of continuously cropped soils, offering an environmentally sustainable and economically viable solution. This study investigated the impacts of distinct straw formulations on the cultivation performance of *Stropharia rugosoannulata* and associated soil microbial communities. Four treatments were established: R group (100% rice straw), C group (100% corn straw), TR group (10% tobacco stalk + 90% rice straw), and TC group (10% tobacco stalk + 90% corn straw). Employing a combined approach of field experiments and high-throughput sequencing, we systematically evaluated treatment efficacy through assessments of agronomic traits and nutritional quality of *S. rugosoannulata*, soil physicochemical parameters, and microbial community profiling. The TC group significantly enhanced *S. rugosoannulata* growth parameters, including height, cap thickness, stipe length, and fresh weight per individual fruiting body, outperforming pure straw treatments. The C group demonstrated the highest crude protein content (32.44%) among all treatments, while nicotine residues in tobacco-containing formulations remained below safety thresholds. Post-harvest incorporation of spent mushroom substrate (SMS) significantly increased soil organic matter (SOM), alkali-hydrolyzable nitrogen (AN), available potassium (AK), and available phosphorus (AP) contents, with the greatest increases observed in the TR group. Notably, the TC group promoted tobacco plant development between 40–80 days post-transplantation, evidenced by significant increases in plant height and leaf area expansion. All treatments substantially reduced incidence rates of tobacco black shank and brown spot disease, with the TR group reducing the incidence by 86.77% and 81.83%, respectively. Microbially, both TC and TR groups significantly enhanced the Chao1 index of soil bacteria and fungi, reduced the Simpson index, and promoted the enrichment of beneficial microbial genera (e.g., *Actinobacteriota*, *Gaiella*, and *Mortierella*), while inhibiting opportunistic pathogenic fungi (e.g., *Neocosmospora* and *Scedosporium*). *β*NTI model analysis revealed that drift and others (DR) primarily governed community assembly patterns, but the TC group uniquely intensified dispersal limitation (DL) effects on fungal community structure.

**IMPORTANCE:**

Driven by the excessive pursuit of economic interests and limited soil resources, the intensive cultivation system centered on continuous cropping has become a critical component of agricultural production. However, this practice has led to soil nutrient depletion, escalated disease pressures, and root decay caused by soilborne pathogens, ultimately hindering healthy crop development. *S. rugosoannulata* cultivation is a non-expensive alternative to enhance soil health. Our study provides the first comprehensive characterization of different straw formulations, demonstrating exceptional potential for enhancing the yield and quality of *S. rugosoannulata*, improving the soil microecology in continuous cropping systems, and suppressing soil-borne diseases. These findings provide a scientific basis for sustainable field management and the recycling of straw resources.

## INTRODUCTION

Soil serves as a fundamental provider of water, nutrients, and thermal conditions during plant growth, playing a crucial role in crop development (1). In Guizhou Province, the majority of the land is characterized by karst topography, resulting in limited availability of arable land resources (2). The issue of soil-borne diseases, a global concern exacerbated by continuous monocropping, has severely restricted the development of mountain agriculture in Guizhou Province (3). The main reason for the decline in crop yield under continuous cropping cultivation is also related to the proliferation of pathogenic fungi, which leads to a decrease in soil fungal diversity and ecological niches (4). Consequently, there is an urgent need for innovative remediation strategies that prioritize environmental sustainability while accounting for inherent soil characteristics—offering fundamental solutions to these challenges.

Biological amendment approaches leverage microorganisms to improve soil physicochemical properties, presenting a cost-effective alternative (5). Such interventions encompass microbial inoculant application, earthworm cultivation, and the planting of salt-tolerant plants, among other ecologically aligned practices (6). Crop rotations, including rotations with different crop varieties, have been proposed to mitigate the negative impacts of pathogens on crop production by breaking the link between plant host and pathogens and becoming a non-expensive alternative for disease control (7). Compared to other biological improvement measures, cultivating edible fungi capable of decomposing crop straws during fallow winter periods offers dual advantages: it provides abundant food resources while generating SMS that effectively enriches soil bioactive compounds (8). Rational straw utilization reduces chemical fertilizer dependency, enhances SOM content, improves overall soil quality, and contributes to the sustainable development of agricultural production (9). *S. rugosoannulata* cultivation primarily utilizes corn cobs, cottonseed hulls, and wheat bran as substrates. Most of these are used for mycelial growth and respiration, while some remain in new forms within the cultivation medium (10). The SMS exhibits favorable physicochemical properties: low bulk density, loose structure, and excellent aeration. Rich in nutrients, microorganisms, and enzymatic activities, this byproduct demonstrates multifaceted benefits for improving soil physicochemical properties, microecological balance, nutrient cycling, and plant growth promotion (11). In recent years, numerous researchers have successfully applied SMS for soil improvement, achieving significant remediation outcomes. However, the characteristics defining these soils are largely unknown. Because of this, advancing our mechanistic understanding of how variations in the assembly of biological improvement influence soil microbiomes is important to provide innovative strategies for improving plant health and productivity.

Microbial ecology is critical to agricultural ecosystems, particularly for soil-based mushroom cultivation. In this study, we conducted a series of experiments to evaluate the soil remediation effects of *S. rugosoannulata* cultivation and SMS application in continuous cropping systems. The aims were to (i) assess the feasibility of substituting partial crop straw with tobacco stalks as cultivation substrate, (ii) investigate the soil improvement effects of SMS returned to fields, and (iii) elucidate the mechanisms underlying soil microbial community assembly following SMS application.

## MATERIALS AND METHODS

### Sample source

**Fig 1:**
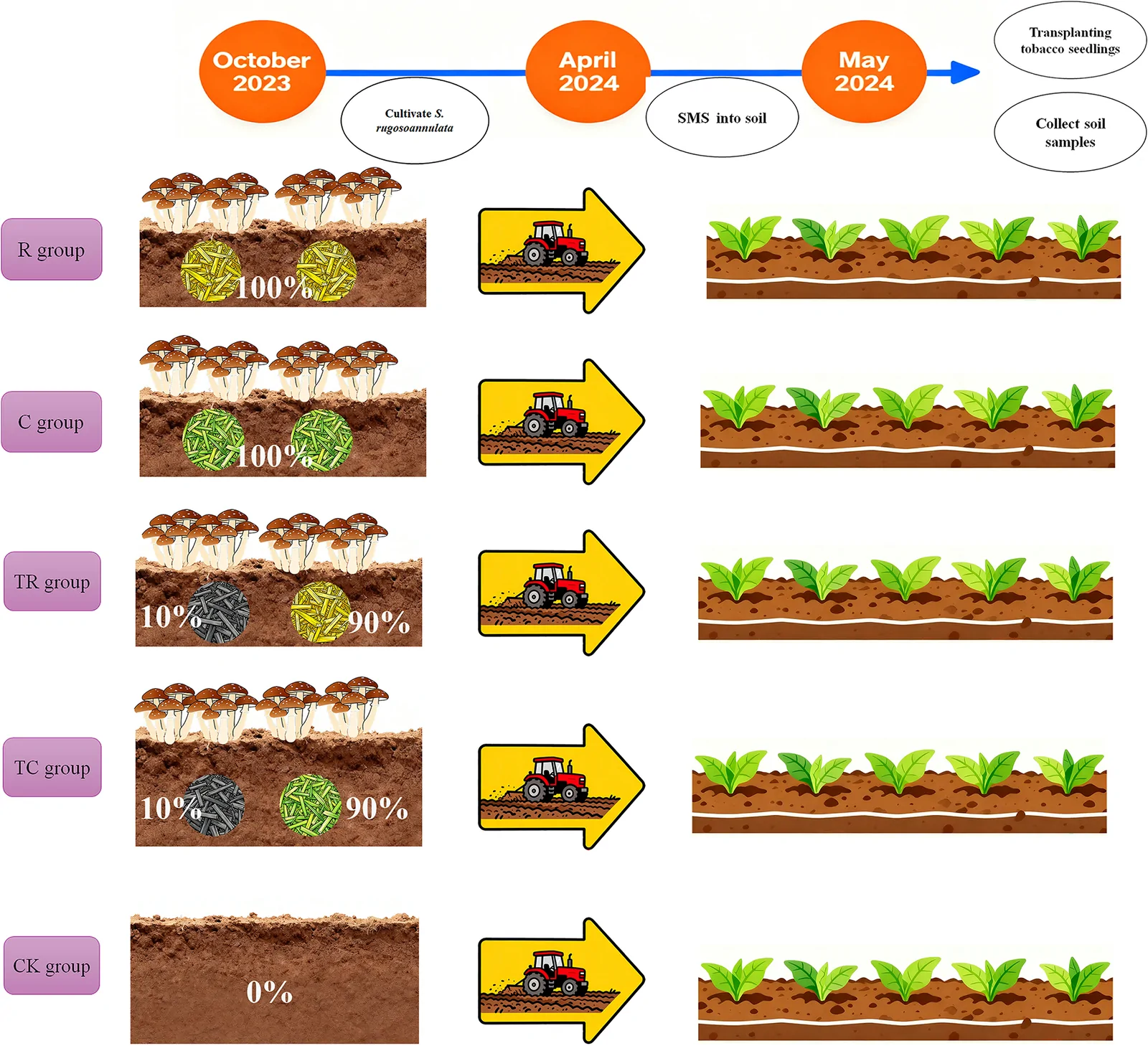
Schematic diagram shows the five cultivation patterns. R group: 100% rice straw; C group: 100% corn straw; TR group: 10% tobacco stalk + 90% rice straw; TC group: 10% tobacco stalk + 90% corn straw.

This study was conducted at the Xingle Technology Park of Zunyi Tobacco Company, Zunyi City, Guizhou Province, China (107°34′ E, 27°40′ N). The soil type was yellow earth, with an average elevation of 1,388 m. Climatic conditions included a mean annual temperature of 14.5°C, 279 frost-free days, and an annual precipitation rate of 1,010 mm. Experimental fields had undergone 10 consecutive years of tobacco monoculture prior to initiation. This study consisted of three consecutive stages: (i) cultivation of *S. rugosoannulata* using four straw formulations, (ii) SMS was incorporated into the soil; and (iii) tobacco planting to assess soil improvement effects (Fig. 1). The field experiment included four cropping treatments: R group (100% rice straw), C group (100% corn straw), TR group (10% tobacco stalk + 90% rice straw), and TC group (10% tobacco stalk + 90% corn straw). Adjacent fields uncultivated with *S. rugosoannulata* served as the control group (CK). Each treatment applied 35 kg/m² of straw substrate. Straw samples were taken from the experimental base. After crops were harvested, straws (rice, corn, and tobacco) that were approximately 10 cm long were screened and sampled, subsequently air-dried, and stored for backup. A 30 cm-thick uniform layer of straw was spread over the soil surface. The spawn of *S. rugosoannulata* was then inoculated into the straw matrix. Following this, a 3–5 cm thick layer of soil was spread evenly over the straw surface to completely cover it. *S. rugosoannulata* was cultivated on 16 October 2023, with fruiting body harvesting occurring from March to April 2024. Following mushroom harvest, SMS residues were mechanically crushed and then evenly incorporated into the soil. Soil samples were collected on 5 May 2024. For each field, the five-point sampling method was used to collect soil samples at a depth of 0–10 cm. Subsequently, each soil sample was divided into two portions: one was placed at 4°C for soil physicochemical measurements, and the other was stored at −80°C for molecular experiments. Fifty-day-old seedlings at the six-leaf stage, which were uniform in size and vigor, were selected for transplantation on 15 May 2024. Commonly used management practices, including tillage, fertilizer application, and weed control, were consistent across all experimental sites.

### Agronomic traits and nutritional quality of *S. rugosoannulata*

Agronomic traits (e.g., cap diameter, cap thickness, and stipe length) of *S. rugosoannulata* fruit bodies were measured using vernier calipers. Crude protein content was determined via the Kjeldahl method (12). For crude fat analysis, samples underwent Soxhlet extraction with petroleum ether; the dried extract was weighed to quantify lipid content (13). Crude fiber was calculated as the high-temperature ashing loss following acid digestion and subsequent acetone removal of ether-soluble fractions. Ash content was expressed as the percentage of residual mass after igniting samples in a muffle furnace until constant weight. Total sugar and polysaccharide contents were assessed using the phenol-sulfuric acid colorimetric assay: samples underwent acid hydrolysis to convert water-soluble and insoluble polysaccharides into reducing sugars, which reacted with phenol for spectrophotometric quantification against a standard curve (14). Reducing sugar content was quantified using 3,5-dinitrosalicylic acid solution (15). Nicotine content was analyzed via high-performance liquid chromatography with UV detection (HPLC-UV) following alkalization and organic solvent extraction of pretreated samples (16).

### Agronomic traits and disease incidence of plants

On the 40 d and 80 d after tobacco transplantation, plant height, stem diameter, number of effective leaves, and maximum leaf area from each plot (15 plants total per plot) were recorded. The disease incidence, including tobacco black shank and brown spot, was investigated based on observations of typical wilt symptoms of leaves on the 120 d.

### Soil physicochemical property analysis

Soil physicochemical properties were determined according to “Soil and Agricultural Chemistry Analysis” (17). pH was measured potentiometrically using a pH meter in a 1:2.5 soil-to-water (w/v) suspension. Soil organic matter (SOM) was determined via potassium dichromate oxidation with external heating. Alkali-hydrolyzable nitrogen (AN) was assessed using the alkaline hydrolysis-diffusion method, titrated with 0.01 M sulfuric acid standard solution. Available phosphorus (AP) was quantified by the molybdenum-antimony colorimetric method. Available potassium (AK) was measured using the flame photometric method.

### Sequencing analysis of soil

Microbial genomic DNA was extracted from soil samples (0.5 g) using a FastDNA SPIN Kit according to the manufacturer’s instructions. The primers 338F (5′-ACTCCTACGGGGAGGCAGCAG-3′)/806R (5′-GGACTACHVGGGTWTCTAAT-3′) and ITS1F (5′-CTTGGTCATTTAGAGGAAGTAA-3′)/ITS2R (5′-GCTGCGTTCTTCATCGATGC-3′) were used to amplify the V3–V4 region of the bacterial 16S rRNA gene and the ITS1 region of the internal transcribed spacer (ITS) region of fungi, respectively. The PCR products were separated by electrophoresis on 1% agarose gel. Raw fastq files were quality-filtered using QIIME33. Low-quality sequences (<150 bp long, with an average quality score <25) were removed. Bacterial and fungal taxonomies were assessed against the 16S rRNA database (Silva v138) and the fungal ITS database (UNITE v7.2), respectively (18).

### Statistical analyses

All statistical analyses were performed in the Majorbio cloud platform and R environment (v4.3.2, http://www.r-project.org/). The taxonomic richness and diversity (alpha diversity) were estimated using the Chao1 and Simpson indices. Based on the Bray–Curtis distance, beta diversity was calculated through non-metric multidimensional scaling (NMDS) to determine the similarities or differences in microbial communities (19, 20). To identify biomarkers among treatments, linear discriminant analysis effect size (LEfSe) analysis was performed with a significance threshold of 0.05 for the factorial Kruskal-Wallis test and an linear Ddiscriminant Aanalysis (LDA) score >3.0. The data were collated and preliminarily mapped using igraph, lavaan, ggplot2, dplyr, and the vegan package in the R 4.3.2 environment. GraphPad Prism 8 and draw.io software were used for plotting.

## RESULTS

### Agronomic traits of *S. rugosoannulata*

**Fig 2:**
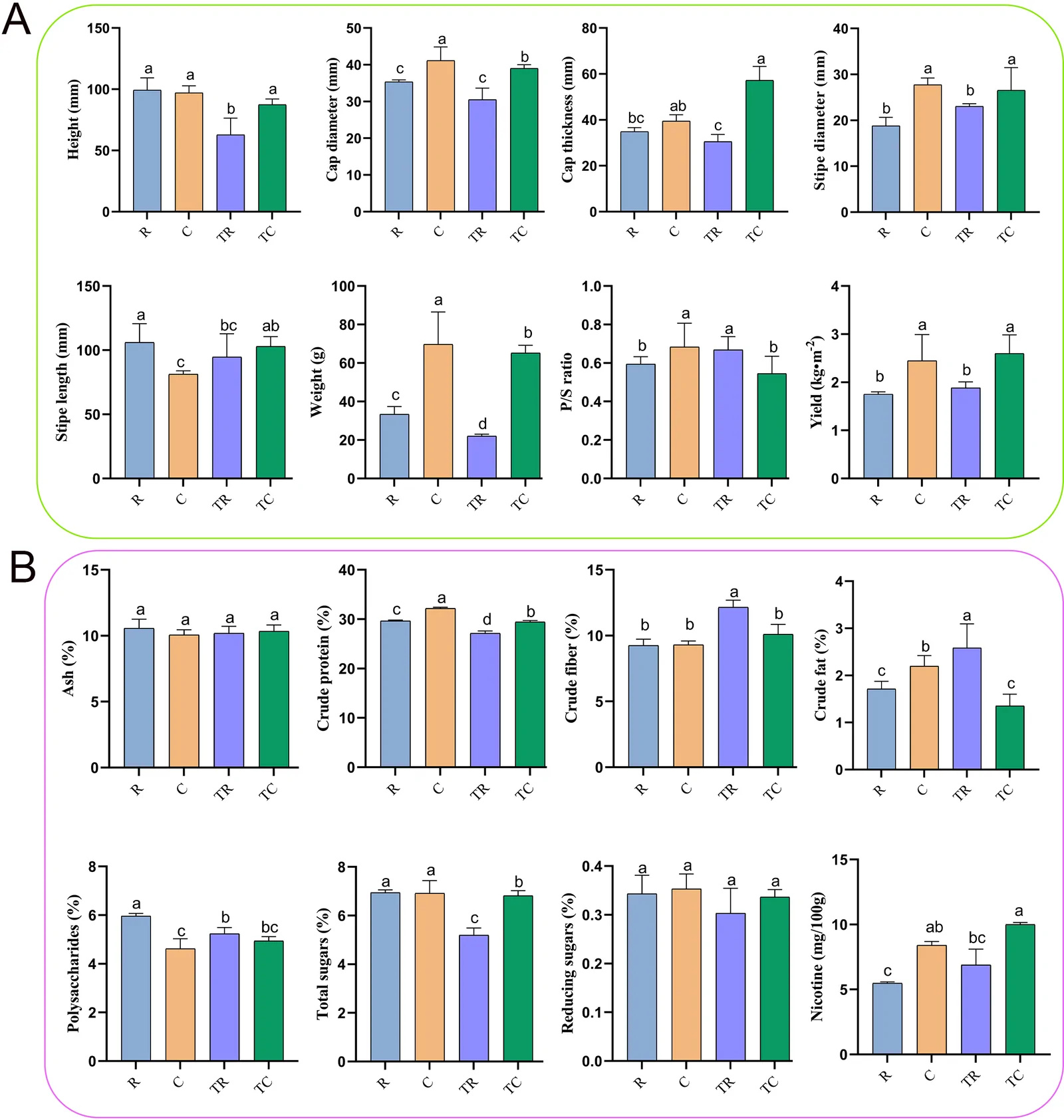
Effects of different straw formulations on the agronomic traits (**A**) and nutritional quality (**B**) of *S. rugosoannulata*. Data are presented as the mean ± standard error (*n* = 3), with significant differences indicated by different letters in the same column at *P* < 0.05.

In this study, four different straw formulations were employed for *S. rugosoannulata* cultivation. Regarding agronomic traits, the fruiting bodies in the R and C groups exhibited the longest overall length, while the TR group showed the shortest height (Fig. 2A). The cap thickness in the TC group (31.72 mm) was significantly greater than in all other groups. The R group recorded the longest stipes (108.86 mm), followed closely by the TC group (107.83 mm), while the C group had the shortest stipes (76.85 mm). Marketable edible mushrooms typically exhibit an optimal mushroom morphology index (*P*/S ratio) within the range of 0.45–0.65. Among the treatments, TC achieved the optimal commercial quality. Additionally, the individual fruiting bodies in the C group had the highest fresh weight (68.85 g), with a yield of 2.59 kg/m². These findings indicated that appropriate incorporation of tobacco stalk into substrate formulations significantly enhanced height, cap thickness, stipe length, and individual fruiting body weight of *S. rugosoannulata*.

### Nutritional quality of *S. rugosoannulata*

This study evaluated the impact of different straw formulations on the nutritional composition of *S. rugosoannulata*, focusing on key quality indicators including crude ash, crude protein, crude fiber, crude fat, polysaccharides, total sugars, reducing sugars, and nicotine (Fig. 2B). The C group exhibited the highest crude protein (32.44%) and total sugar content (7.25%). The highest polysaccharide content was found in the R group (5.97%), while the lowest was in the C group (4.69%). The C group also had the highest total sugar content (7.25%), while the TR group had the lowest (5.19%). Nicotine concentration correlated positively with the proportion of tobacco stalks: the TR group contained higher nicotine than the R group, and the TC group exceeded the C group. Crucially, the absence of significant variations in nicotine content supports the safety of using tobacco stalk as a substitute substrate component for *S. rugosoannulata* cultivation in practical agricultural settings.

### Soil physicochemical property analysis

**Fig 3:**
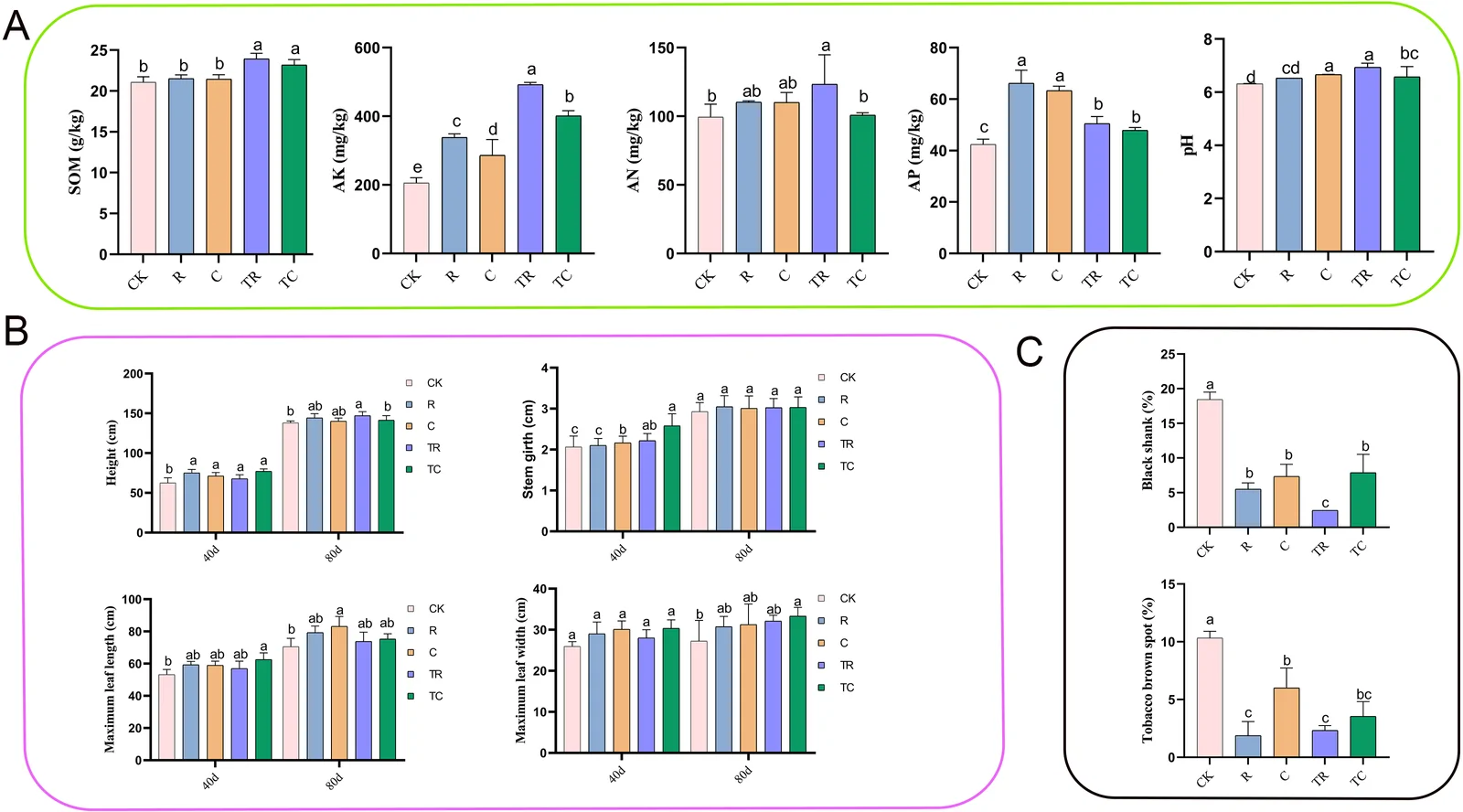
Effects of different straw formulations on soil physical and chemical properties (**A**), tobacco agronomic traits (**B**), and disease incidence (**C**). Data are presented as the mean ± standard error (*n* = 3), with significant differences indicated by different letters in the same column at *P* < 0.05. SOM, soil organic matter; AK, available potassium; AN, alkali-hydrolyzed nitrogen; AP, available phosphorus.

To understand the influence of different straw formulations on the soil environment, the variations in the soil physicochemical characteristics in the different experimental fields were compared (Fig. 3A). The contents of SOM, AK, AN, and pH were the highest in the TR group, followed by the R and TC groups (*P* < 0.05). Similarly, compared to the CK group, the AP content was significantly increased in all treatment groups (*P* < 0.05), with improvements of 55.82%, 49.04%, 18.91%, and 12.88%, respectively.

### Agronomic traits and disease incidence of plants

At 40 days post-transplantation, tobacco plants in the TC group exhibited better growth metrics, including significantly greater plant height, stem girth, maximum leaf length, and leaf width compared to the CK group (Fig. 3B). At 80 d, the TC group plants maintained robust development with an average height of 141.6 cm. Notably, straw formulations exerted pronounced effects on early-stage tobacco growth, with the TC group particularly enhancing rapid expansion of plant height and leaf area during the initial phase. However, by the vigorous growth stage, inter-treatment differences diminished, and overall growth uniformity increased. Incorporation of SMS into soil significantly reduced tobacco disease incidence (Fig. 3C). All treatments showed significantly lower tobacco black shank disease incidence compared to CK (*P* < 0.05), with the TR group achieving an 86.77% reduction. Similarly, tobacco brown spot incidence was significantly suppressed across all treatments, showing respective reductions of 81.83%, 41.91%, 77.41%, and 65.69% compared to CK.

### Soil microbial community structure and composition

**Fig 4:**
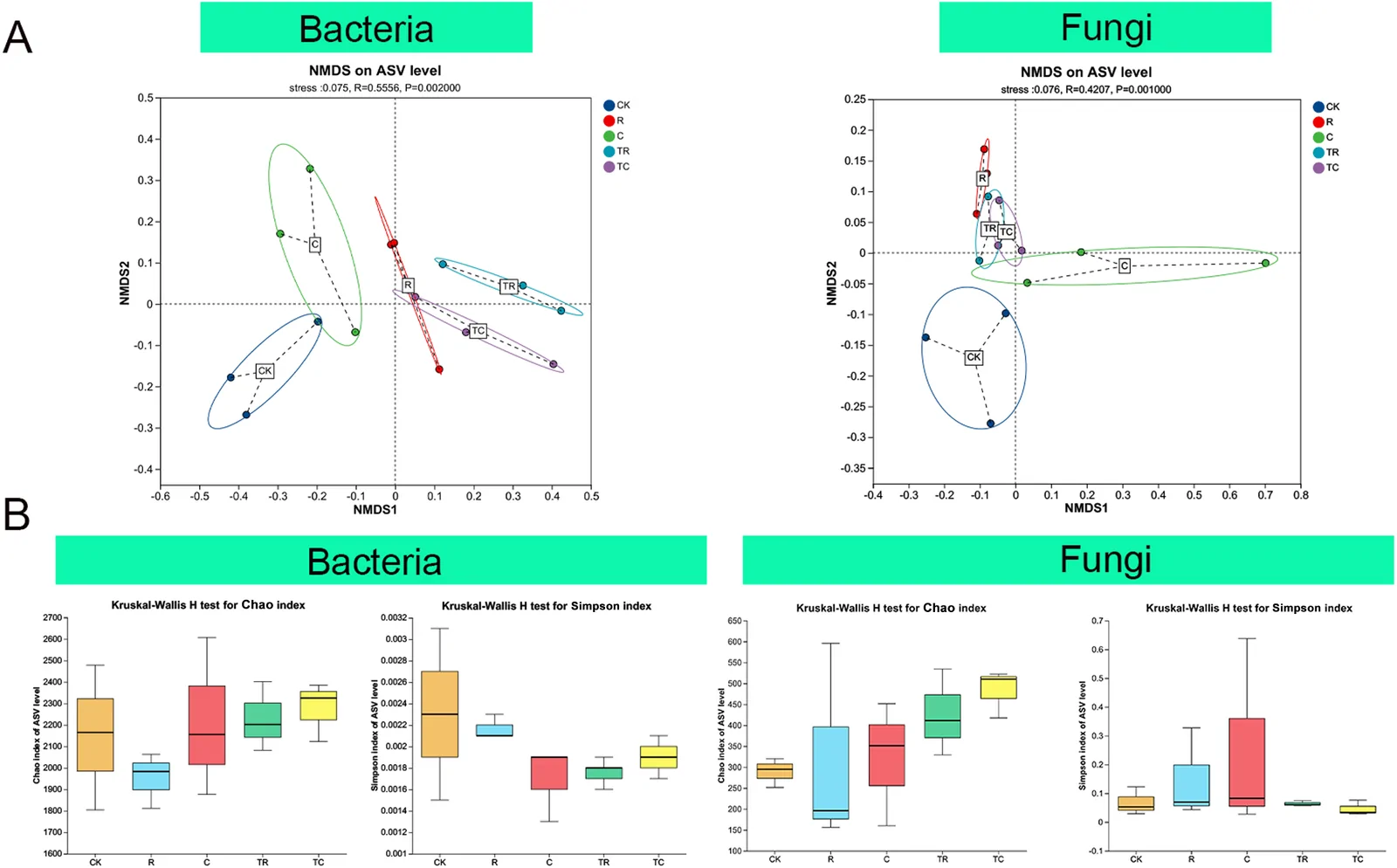
Changes in microbial community in soil. (**A**) Non-metric multidimensional scaling (NMDS) of microbial communities in soil bacterial and fungal community structures. (**B**) The Chao1 and Simpson diversity indices of the bacteria and fungi in the soil.

To explore the changes in soil microbial communities after cultivating *S. rugosoannulata* with different straw formulations, we performed NMDS analysis at the amplicon sequence variant (ASV) level for both bacterial and fungal communities (Fig. 4A). The bacterial microbiome plays a crucial role in influencing the severity of soil-borne diseases compared to fungi. Healthy plants typically harbor bacterial communities with greater diversity and richness, which effectively inhibit pathogen invasion, particularly for fungal pathogens (21). In this study, significant differentiation among treatments was observed for both bacteria (R² = 0.5556, *P* = 0.0020) and fungi (R² =0.4207, *P* = 0.0010). The replicates of each treatment clustered together, indicating minimal variation within treatments. Following SMS application, soil bacterial and fungal community structures exhibited distinct grouping patterns across treatments. These findings collectively underscore that differential SMS amendments profoundly shape soil microbiome architecture.

Sequentially, we compared the variation of bacterial and fungal communities between different straw formulations. The Chao1 index reflects the species richness of the soil microbial community. For bacterial communities, the TC group had the highest Chao1 index (2,277), indicating the greatest species richness (Fig. 4B). This was followed by the TR group and C group. Notably, the R group showed the lowest richness (1,952) among formulated groups but still exceeded the CK (2,149). These results suggest that the mixed straw formulations containing tobacco stalks (TR, TC) are more favorable for microbial species richness. The Simpson index reflects the degree of dominance by the species within a community, with a lower value indicating higher diversity. The C group achieved the lowest Simpson value (0.0017), followed by the TR group (0.0018) and the TC group (0.0019). For fungi, the TC group exhibited the highest fungal Chao1 index, followed by the TR group, while the R and C groups were similar, slightly higher than the CK. The Simpson index for the TC treatment group was the lowest. The R group and the C group had relatively higher values. In conclusion, for enhancing soil fungal diversity, either the TC or TR group is recommended for *S. rugosoannulata* cultivation, as they improve the microbial environment of monoculture soils.

**Fig 5:**
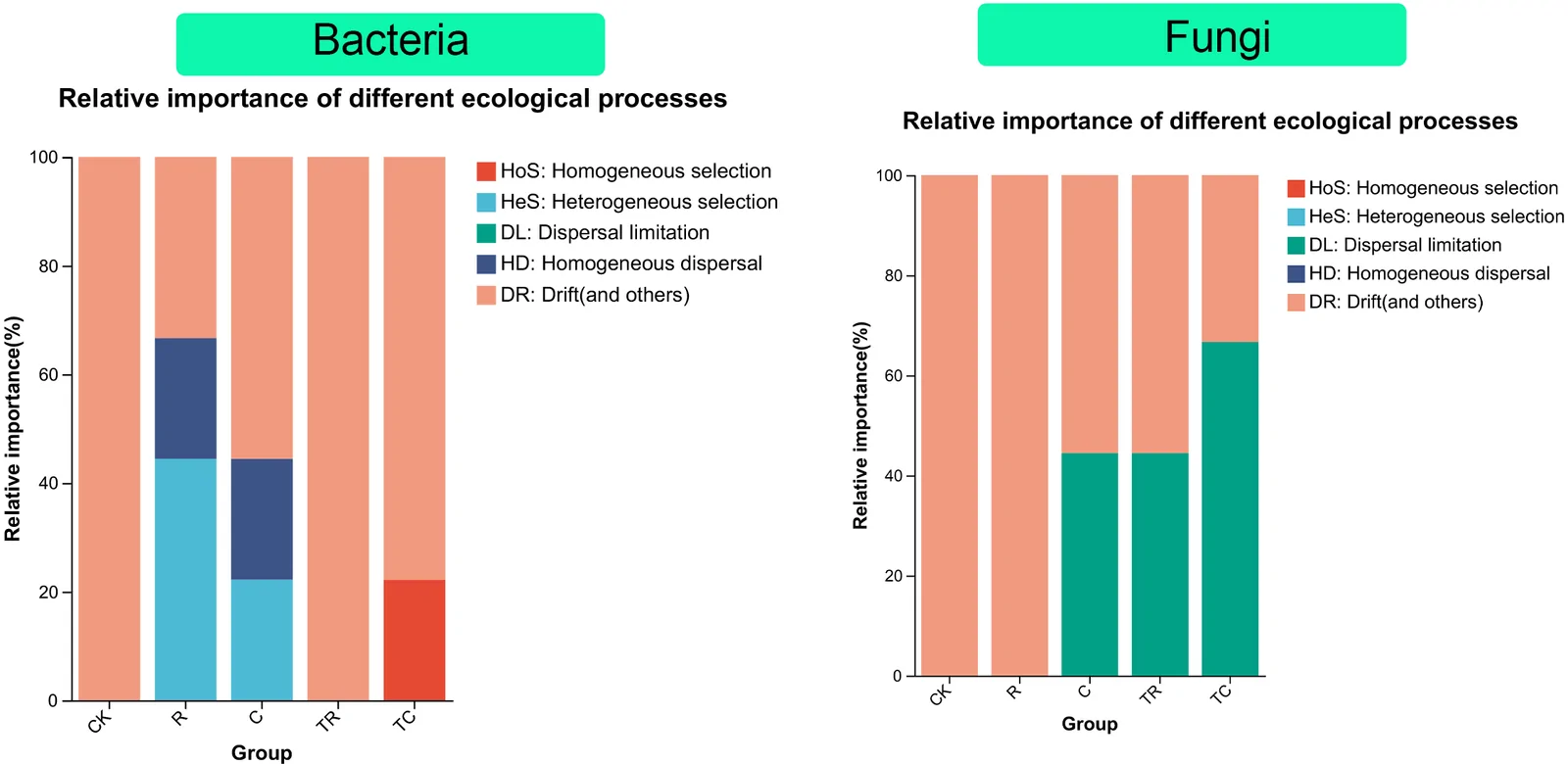
Ecological processes shaping bacterial and fungal communities in different experimental fields through *β*NTI model.

Furthermore, this study employed null model analysis (999 randomizations) to calculate the *β* nearest taxon index (βNTI) and assess the relative importance of deterministic and stochastic processes in microbial community assembly (Fig. 5) (22). For bacterial communities, the results revealed that stochastic processes dominated microbial community assembly across all treatment groups. In fungal communities, drift and others (DR) similarly dominated in both the CK and R groups. In the C group, similar to the TR group, DR accounted for approximately 55%, while dispersal limitation (DL) contributed about 45%. Notably, the TC group demonstrated a striking shift: DR decreased to 35%, while DL surged to 65%, indicating that the corn straw and tobacco stalk substantially amplified the influence of DL, elevating it to a primary driver of fungal community assembly.

**Fig 6:**
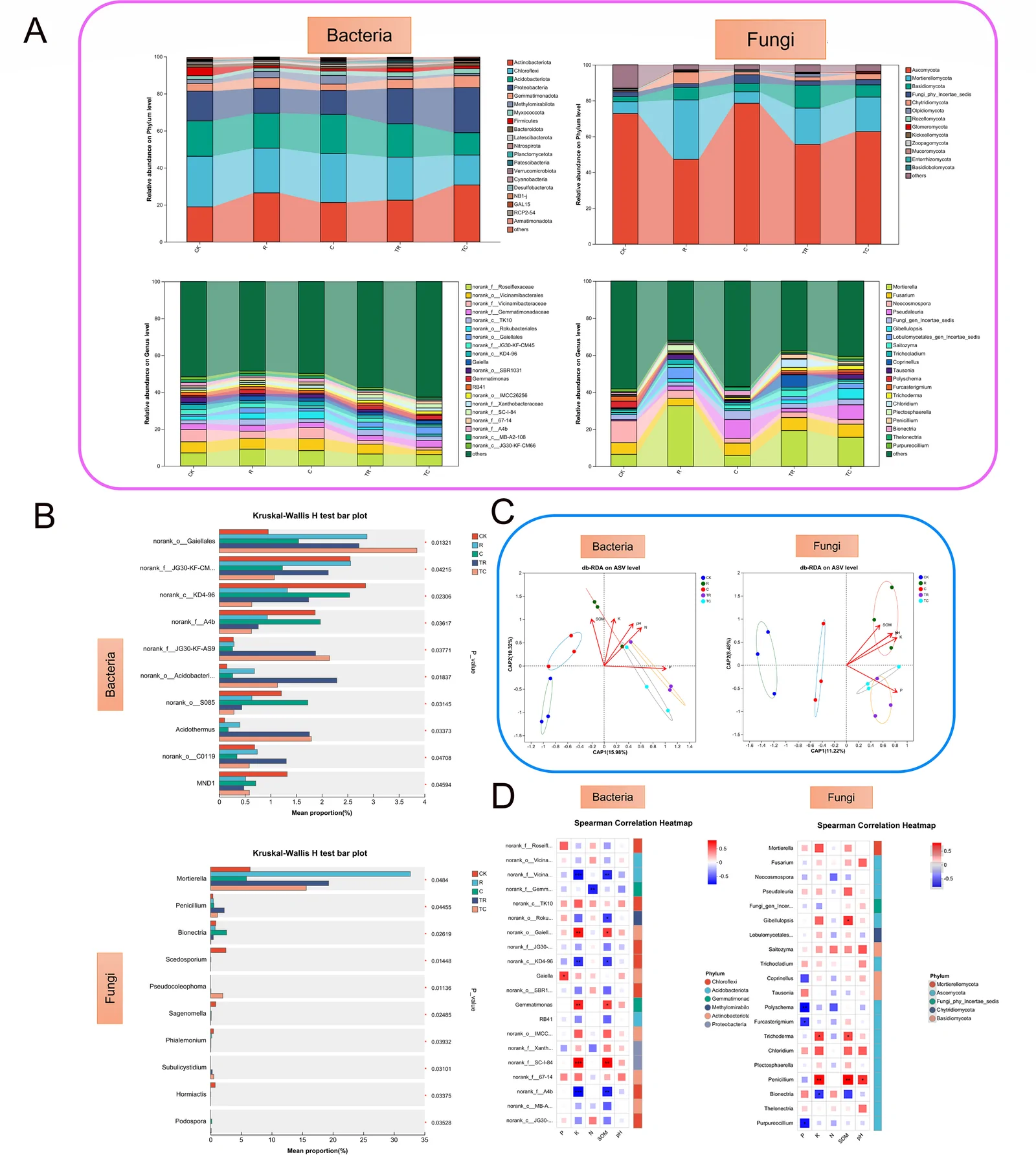
Effects of different straw formulations on the soil microbial community with correlations of soil physicochemical variables in the cultivated soil of *S. rugosoannulata*. (**A**) Effect of different straw formulations on the soil microbial community composition at the phylum and genus levels. (**B**) Indicator bacterial and fungal taxa in different experimental fields. (**C**) Distance-based redundancy analysis at the ASV level, different colors represent different treatments. (**D**) Correlation heatmap of the top 20 bacterial and fungal genera with the environmental factors. SOM, soil organic matter; K, available potassium; N, alkali-hydrolyzed nitrogen; *P*, available phosphorus. The R values are indicated on the right side of the legend with different colors. *P < 0.05, **P < 0.01, and ***P < 0.001.

At the bacterial phylum level, the CK was dominated by Chloroflexi (27.31%) and Acidobacteriota (19.12%) (Fig. 6A). Notably, Actinobacteriota reached its peak abundance in the TC group (30.7%), representing a 62.4% increase over CK. Proteobacteria likewise showed significant enrichment in the TC group (24.3%). Conversely, Acidobacteriota exhibited a declining trend with SMS application, dropping to 12.0% in the TC group. At the genus level, *Roseiflexaceae* (7.08%) and *Vicinamibacterales* (6.02%) were dominant in the CK group. *Roseiflexaceae* reached maximum abundance in the R group (9.1%) and minimum in the TC group (6.2%). Gaiellales increased 4.1-fold (3.9%) in the TC group compared to CK. The TR group also significantly enriched *Gaiella* and *Gemmatimonas*, suggesting synergistic stimulation of beneficial genera by corn straw-tobacco stalk formulations. At the phylum level of the fungal community, Ascomycota dominated in all treatments, but with significant differences in relative abundance (Fig. 6B). Ascomycota was dominant in CK (72.8%) and the C group (78.6%), yet decreased progressively in the R, TC, and TR groups, reaching the lowest value of 47.3% in the R group. Mortierellomycota reached its highest value in the R group at 33.1%, significantly higher than CK (6.59%, *P* < 0.01); the TC and TR groups also reached 19.2% and 20.2%, respectively. Basidiomycota was highest in the TR group at 12.8%, significantly higher than CK (2.86%). At the genus level, the dominant genus structure was significantly altered across treatments. In the R group, the relative abundance of *Mortierella* reached 32.68%, making it the absolute dominant genus. In the C group, the proportion of *Pseudaleuria* reached 10.16%, with *Coprinellus* and *Chloridium* emerging as dominant genera in the TR group.

**Fig 7:**
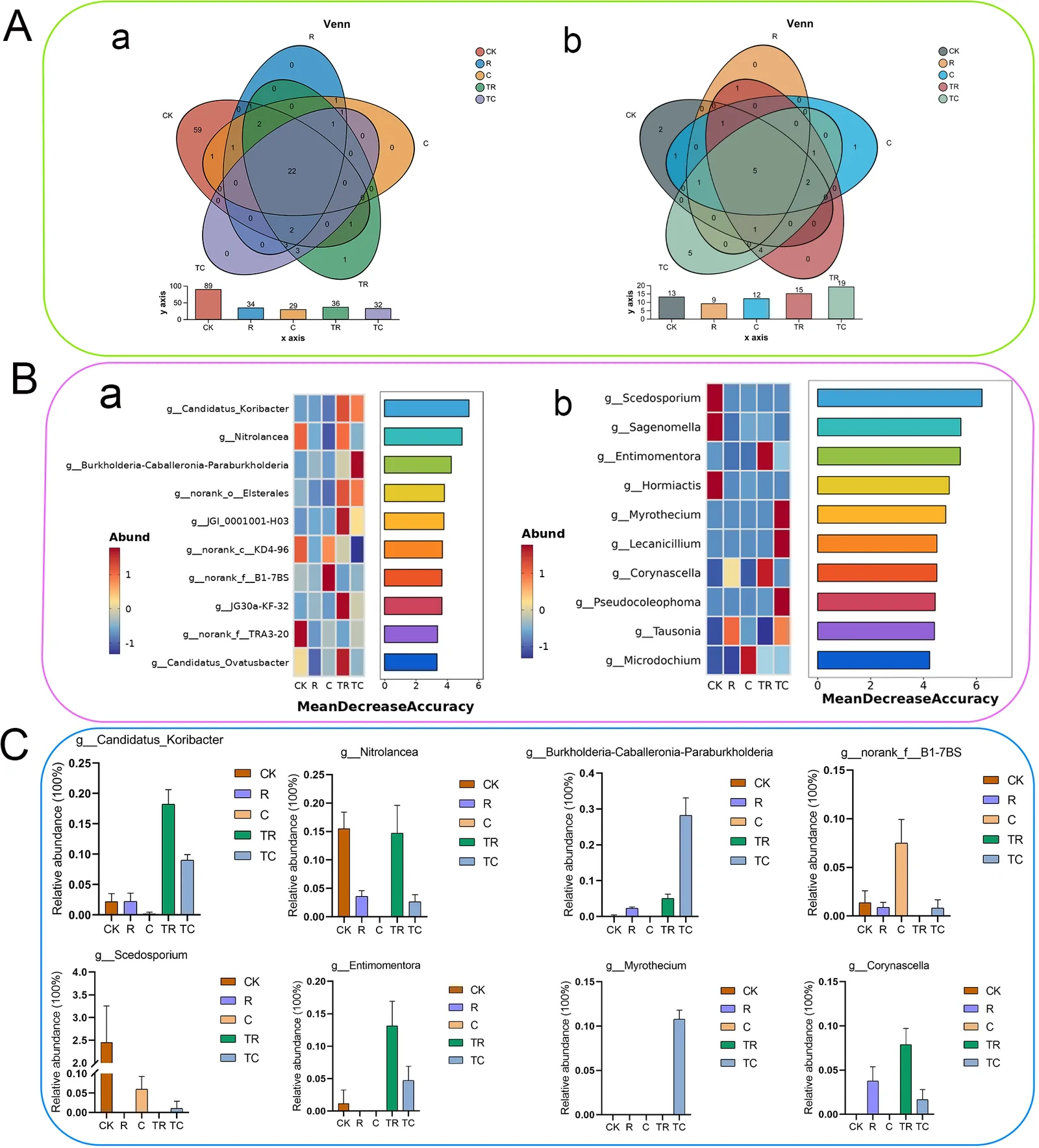
Similarity of abundance shifts in microbiomes driven by treatments and relative abundances of keystone genera. (**A**) Venn diagram of differentially abundant genera identified by LEfSe analysis. (**B**) Relative abundances of keystone genera identified by a combination of LEfSe and random forest analysis. (**C**) Relative abundances of the single most abundant biomarkers in different treatments.

The taxonomic and functional convergence of microbiomes pointed out that specific species in microbiomes affected by treatments could functionally modify microbiome activity (23). The biomarkers provided a representative snapshot of soil microorganism composition (4). LEfSe analysis (LDA > 3.0) revealed that most genera with abundance shifts were shared among each treatment (Fig. 7A). To reduce the time and cost for simplified microbiome construction, community metabolic modeling was used to model community functions and simulate performances of alternative community combinations. To this end, we used random forest analysis to identify the key genera (top 10) among the significantly changed genera identified by LEfSe. For bacteria, the TR and TC treatment groups, the number of different biomarkers was significantly higher than that in the other groups (Fig. 7B). *norank_f__B1-7BS* was present in the C group. The TR group contained six types of biomarkers, such as *Nitrolancea*, *JGI_0001001-H03*, *JG30a-KF-32,* and *Candidatus_Ovatusbacter*. The TC group contained three biomarkers, such as *Burkholderia-Caballeronia-Paraburkholderia*. For the fungal communities, *Tausonia* and *Microdochium* were identified as biomarkers for the R group and the C group. *Entimomentora* and *Corynascella* were identified as biomarkers for the TR group. The TC group contained three biomarkers, such as *Myrothecium*, *Lecanicillium*, and *Pseudocoleophoma*.

### Correlation analysis between soil microbial communities and physicochemical properties

To disentangle relationships between edaphic factors and microbial communities, we employed distance-based redundancy analysis (db-RDA) at the ASV level (Fig. 6C). For bacterial communities, the first constraining axis (CAP1) explained 15.98% of the variance, while the second axis (CAP2) accounted for 13.18%. Among the soil factors, soil AP had the greatest impact on bacterial community composition, followed by AK. AN and SOM had a smaller impact on sample distribution. In the fungal community, CAP1 explained 11.22% of the variation, and CAP2 explained 8.48%. AP, AN, and pH had the greatest impact on the fungal community structure, with additional effects from the accumulation of AK and SOM.

A Pearson correlation analysis was performed to investigate the influence of soil physicochemical properties on the dominant soil species. From Fig. 6D, it is evident that for the bacterial community, SOM was significantly positively correlated with *Gemmatimonas* and *Gaiellales*, and significantly negatively correlated with *Vicinamibacteraceae*. AN demonstrated a significant negative relationship with *Gemmatimonadaceae*. AP positively correlated with *Gaiella*, while AK showed positive associations with both *Gaiellales* and *Gemmatimonadaceae* alongside a negative correlation with Vicinamibacteraceae. For fungal communities, AP negatively correlated with *Polyschema*, *Furcasterigmium*, and *Purpureocillium*. AK positively correlated with *Penicillium* and *Trichoderma* but negatively with *Bionectria*. SOM displayed positive relationships with *Penicillium*, *Trichoderma*, and *Gibellulopsis*, while pH positively correlated with *Penicillium*.

**Fig 8:**
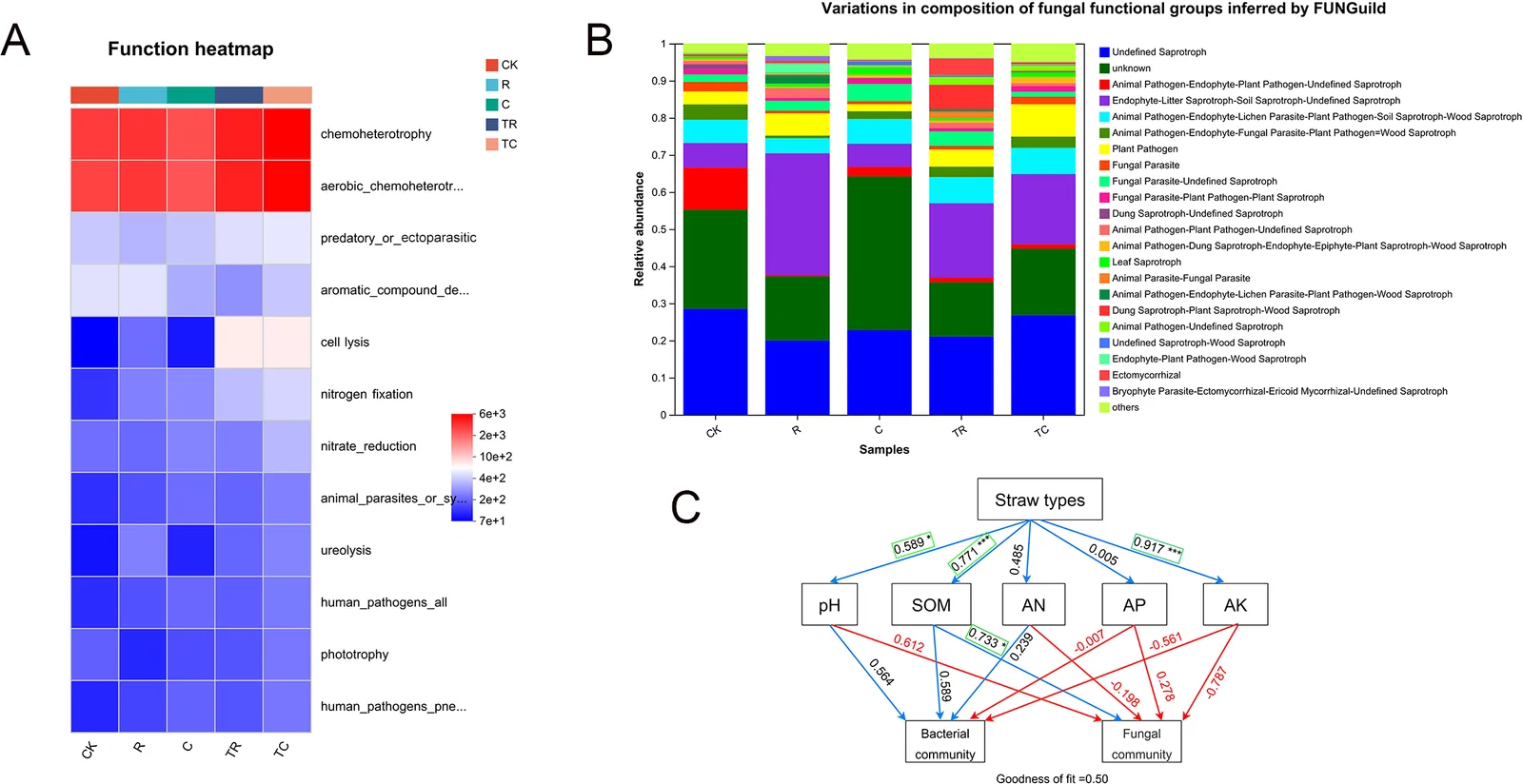
Different straw formulations for cultivating *S. rugosoannulata* and analyzing their relationship with the soil microbial community, based on the FAPROTAX database analysis (**A**), FUNGuild database analysis (**B**), and structural equation modeling analysis (SEM) (**C**). The numbers adjacent to the arrows represent path coefficients, with blue and red arrows indicating positive and negative effects, respectively. SOM, soil organic matter; AK, available potassium; AN, alkali-hydrolyzed nitrogen; AP, available phosphorus. Significance levels are indicated as *P < 0.05 and ***P < 0.001.

Structural equation modeling (SEM) was conducted to investigate the correlation between different straw formulations and the soil microbial community (Fig. 8C). The results showed that the straw types were significantly positively correlated with pH, SOM, and AK. SOM had a positive effect on the fungal community. Thus, SOM had the greatest effect on the fungal community.

### Soil microbial function prediction

FAPROTAX functional prediction analysis was employed to investigate the functional changes in bacterial communities under different treatments (Fig. 8A). Chemoheterotrophy and aerobic chemoheterotrophy collectively constituted over 70% of predicted functions, making them the dominant bacterial functions. Notably, the enrichment of genera such as *Burkholderia-Caballeronia-Paraburkholderia* and *Gaiella* directly contributes to these dominant chemoheterotrophic processes in the TC group, which are central to the decomposition of complex organic matter (e.g., cellulose and lignin) from the corn-tobacco straw and the cycling of soil carbon (24–26). The relative abundance of cellulolysis increased in both TR and TC groups compared to CK, indicating enrichment of bacterial taxa involved in cellulose decomposition.

To clarify the functional changes in the soil fungal community, FUNGuild functional prediction analysis was applied (Fig. 8B). The Undefined Saprotroph had the highest abundance (13.52%) in the TC group, which was significantly higher than that in the CK. Wood Saprotrophs surged in TC and TR groups by 3.4-fold and 2.3-fold over CK, respectively. This functional enrichment is directly attributable to recalcitrant lignocellulose derived predominantly from corn straw, which provides the essential substrate that selectively enriches fungal taxa capable of decomposing complex woody polymers. Concurrently, the relative abundance of arbuscular mycorrhizal fungi (AMF) in the TC treatment increased to 3.62%, a 9.5-fold enhancement compared to CK. The significant enrichment of AMF is of particular agronomic relevance in continuous cropping systems, as it enhances plant phosphorus acquisition and improves tolerance to environmental stresses, thereby contributing to the observed suppression of soil-borne diseases and promotion of plant growth. Animal pathogen-plant pathogen abundance in the CK reached 57.3%, while it decreased by more than 50% following SMS application, which directly reduced the pathogenic pressure associated with continuous cropping.

## DISCUSSION

### Straw formulation optimizes *S. rugosoannulata*’s yield and nutritional quality

In the current study, we established 100% rice straw (R) and 100% corn straw (C) as “pure straw” controls, and the TR and TC groups with 10% tobacco stalk substitution as experimental treatments; we conducted multidimensional tracking analysis. Among all groups, the C group exhibited superior performance in cap diameter (40.67 mm), crude protein content (32.44%), and individual fresh weight (68.85 g), indicating that the high C/N ratio of corn straw promotes the expansion of fruit bodies and nutrient accumulation (24). The TC group maintained the high-yield advantage of the C group and also showed increased cap thickness with an optimal mushroom morphology index, exhibiting a synergistic effect of high yield and superior commercial quality. The pure rice straw group (R) showed lower cap thickness and individual fresh weight compared to mixed-substrate groups, suggesting that the singular nutrient profile of pure rice straw limits yield. The excellent agronomic traits in the TC group are mechanistically linked to the synergistic decomposition of the mixed substrate. Corn straw provided an available carbon source for rapid mycelial colonization, while tobacco stalk contributed to prolonged nutrient release.

### Soil property improvements driven by SMS amendment

Cultivation of *S. rugosoannulata* significantly enhanced soil fertility. Soil physicochemical analysis revealed distinct substrate-dependent impacts: the C group exhibited significantly higher SOM and AK contents than the R group, while registering a lower pH value. This observation suggested that corn straw decomposition demonstrates weaker inhibition of soil acidification compared to rice straw. The diminished AN content observed in the R group may be attributed to nitrogen immobilization stemming from rice straw’s elevated carbon-to-nitrogen (C/N) ratio (27). Notably, AP, AK, and pH in all treatment groups significantly increased compared to the CK group.

The significant increases in SOM, AN, AP, and AK following SMS incorporation are a direct consequence of microbial transformation. The FAPROTAX prediction confirmed the dominance of chemoheterotrophy and a significant increase in cellulolysis in the TC and TR groups. The integration of microbial taxonomy with functional predictions provides a mechanistic understanding of soil remediation. The dominance of chemoheterotrophy, driven by the enrichment of bacterial genera like *Burkholderia-Caballeronia-Paraburkholderia* and *Gaiella* in the TC group, highlights a stimulated microbial metabolism geared towards the corn and tobacco-straw substrates. Meanwhile, a positive correlation was observed between soil AP content and the oligotrophic genus *Gaiella*, suggesting that *Gaiella* may be involved in phosphorus cycling and potentially contribute to the elevated soil AP content.

### Response of soil microbial community structure to straw formulation

Rice-mushroom rotation markedly increased the relative alpha diversity index of soil bacteria and enriched beneficial microbiota (28). *Neocosmospora* abundance in CK exceeded all mushroom-amended treatments, suggesting that SMS incorporation mitigates soilborne pathogen pressure via microbial antagonism or induced systemic resistance (29). *Gaiellales*, carrying cellulase genes, were substantially enriched in the C group, likely due to corn straw’s high lignocellulose content (30). For fungi, the *Mortierella* acted as the most prominent rice straw-responsive microorganism, while the abundance of potential pathogens such as *Scedosporium* dropped by over 90% in the R group. The polysaccharide enzymes produced by *Mortierella* effectively decompose cellulose, with metabolic byproducts potentially creating antifungal environments that reduce *Neocosmospora* dominance across treatments (31). The explosive growth of *Mortierella* aligns with previous conclusions that straw incorporation promotes saprophytic fungi proliferation (32).

New bacterial biomarkers, including *Pseudocoleophoma*, *Lecanicillium*, and *Myrothecium*, emerged in the TC group, and *Burkholderia-Caballeronia-Paraburkholderia* became the core bacterial biomarkers, forming unique signatures. The appearance of low-abundance genera like *Myrothecium* and *Lecanicillium* suggested that the TC group formulation contributes most to functional diversity. *Lecanicillium* carried abundant chitinase genes, implying potential biocontrol mechanisms through pathogen cell wall degradation (33, 34).

The significant shift in fungal community assembly of the TC group is attributed to the complex composition of corn straw-tobacco stalk blends that increase soil microenvironmental heterogeneity (35). Potential antimicrobial compounds (e.g., nicotine) from tobacco stalk have increased the spatial heterogeneity and environmental filtering of soil microhabitats (36, 37). This enhanced environmental filtering selectively enriches functional microbes (e.g., arbuscular mycorrhizal fungi and wood saprotrophs) while inhibiting pathogen dispersal, directly supporting the observed soil disease suppression and tobacco growth promotion.

### Microbial-mediated disease suppression mechanisms

Continuous monoculture is frequently accompanied by the enrichment of soilborne pathogens and the depletion of beneficial microbial consortia, which collectively exacerbates disease incidence and threatens crop productivity (22, 36). We found that SMS made from tobacco stalk and crop straw mixtures effectively suppressed diseases. The TC and TR groups performed best, reducing tobacco black shank incidence by up to 86.77% and brown spot by up to 81.83% compared to the CK group. LEfSe analysis (LDA > 3.0) identified unique microbial biomarkers in the TC and TR groups with well-documented biocontrol potentials, which directly inhibit soilborne pathogens through enzymatic degradation or secondary metabolite production. The TC group uniquely enriched nitrogen-cycling biomarkers such as *Burkholderia*, while TR enriched lignin-degrading fungi (e.g., *Entimomentora*). These biomarkers provided critical insights into the mechanistic basis of soil improvement. *Myrothecium* are ubiquitous soil saprophytic fungi that produce and secrete β-glucosidase and xylanase and possess quantifiable lignocellulolytic potential that facilitates the degradation of complex organic matter (e.g., cellulose and hemicellulose) in soil ecosystems (38). *Lecanicillium* has also been reported to be capable of mycoparasitizing fungal pathogens via direct hyphal penetration and enzymatic cell wall degradation, thereby contributing to the suppression of plant pathogens and subsequent promotion of plant health (39, 40). The enrichment of such taxa in the TC group offers a plausible mechanistic rationale for the observed inhibition of soil-borne diseases (e.g., tobacco black shank and brown spot) in the studied system.

The FUNGuild prediction revealed a significant increase in wood saprotrophs in the TC group, suggesting an enrichment of fungi specialized in decomposing recalcitrant organic matter. This enrichment intensifies competition between saprotrophic microbes and soilborne pathogens for limited resources and spatial niches. The dramatic increase in wood saprotrophs, correlated with the degradation of lignocellulose-rich straw, not only accelerates the breakdown of the spent substrate but also facilitates the formation of SOM. This improvement in soil structure enhances aeration and water infiltration, simultaneously reducing the persistence of anaerobic pathogens (41). Furthermore, the decomposition activities of saprotrophs compete directly with soilborne pathogens for resources and spatial niches, effectively imposing a biological suppression. Additionally, the significant enrichment of AMF in the TC group plays a key role in enhancing tobacco disease resistance. AMF form symbiotic associations with tobacco roots, improving plant phosphorus acquisition and inducing systemic resistance against soilborne pathogens (42). Thus, the synergistic action of these groups collectively explains the observed mitigation of continuous cropping obstacles, including enhanced tobacco growth and suppressed disease incidence.

### Changes in the relationship between microbiome and soil properties

Bacterial communities were most significantly influenced by AP, as evidenced by its 15.98% explanatory contribution in the first axis of constrained ordination analysis (CAP1). AP exhibited positive correlations with oligotrophic genera, including *Gaiella*, while negatively correlating with copiotrophic taxa like *Vicinamibacteraceae*, suggesting that SMS application may enhance their phosphorus-solubilizing capacities. In the fungal community, AP was significantly negatively correlated with pathogen-associated genera, including P*olyschema,* illustrating the mechanism of abiotic pathogen suppression through nutrient-mediated environmental filtering. The AP/AK ratio emerged as a critical parameter regulating microbial functionality, offering quantitative guidance for optimizing straw formulations. Community assembly analysis revealed that stochastic processes (DR) dominated across treatments (>60%), yet DL surged to 65% in the TC group, indicating that corn straw and tobacco stalk substantially amplified the influence of DL.

### Effects of tobacco bioactive compounds and straw formulations on soil pH and microbial dynamics

This study was conducted in the representative acidic yellow earth (pH < 6.5) of Guizhou Province, emblematic of karst landscapes in Southwest China where acidic soils and long-term continuous tobacco cropping dominate agricultural systems. Experimental groups incorporating corn straw (C group), tobacco stalk-corn straw (TC group), and tobacco stalk-rice straw (TR group) formulations effectively increased soil pH while significantly enhancing SOM accumulation. This formulation-dependent pH response would perform differently in alkaline soils. In contrast to acidic soils where these formulations enriched beneficial genera (e.g., *Gaiella* and *Mortierella*) and suppressed acidophilic pathogens (e.g., *Neocosmospora*), the buffering capacity of corn straw and tobacco stalks may be neutralized by inherent soil alkalinity in non-acidic contexts, potentially diminishing their competitive advantage in regulating microbial communities (31, 43). In this study, the TC group’s fruiting bodies contained the highest nicotine content (0.841 mg/kg⁻¹), well within safety standards (European Food Safety Authority MRLs of 1.17 mg·kg⁻¹ for dried mushrooms) (44). Environmental persistence of nicotine is limited under field conditions. The observed enrichment of microbial genera with documented pesticide-degrading capabilities (e.g., *Burkholderia* and *Streptomyces*) in our TC and TR treatments further enhances nicotine breakdown (43). These specialized degraders actively mineralize nicotine into harmless metabolic products, preventing environmental accumulation. Beyond nicotine, tobacco stalks contain a suite of other bioactive compounds, including solanesol, polyphenolic allelochemicals, and alkaloids, which are known to exhibit allelopathic or antimicrobial properties (45–48). The robust growth of *S. rugosoannulata*, improved soil health, and enhanced tobacco performance indicate that these compounds are non-phytotoxic to the fungus and contribute to overall disease suppression and microbiome modulation. Future metabolomic profiling is warranted to link the dynamics of these bioactive compounds to observed microbial and agronomic responses, and to assess their potential soil ecological impacts.

This study demonstrated that tobacco stalk-crop straw blends for *S. rugosoannulata* cultivation effectively enhance continuous cropping soil health. After the winter idle season, the SMS was incorporated into soil, overcoming key challenges in traditional straw returning practices, such as the single carbon source input, soil acidification risks, and pathogen proliferation. While the short-term benefits are evident, the long-term sustainability of this approach requires further investigation. The observed increases in SOM and microbial diversity represent initial conditions that may initiate positive feedback mechanisms. SOM provides substrate for stable aggregate formation, potentially leading to improved physical structure and hydraulic properties that support microbial habitat continuity (49). Furthermore, the established beneficial microbial communities, particularly the enriched AMF and biocontrol agents, create the positive legacy effect, leading to sustained nutrient cycling and pathogen suppression in subsequent cropping cycles (49). The tobacco-straw integration model not only utilized tobacco field waste but also synergizes economic gains for farmers with ecological preservation, aligning with the agricultural development goals of “carbon neutrality.” However, these interpretations must be tempered by recognition of the study’s spatial limitation to acidic soils in Guizhou Province and temporal limitation to a single growing cycle. Future investigations should employ multi-year monitoring across diverse edaphic conditions to quantify the persistence of observed effects and their impacts on soil carbon sequestration potential.

### Conclusion

This study systematically elucidated the regulatory mechanisms of straw formulations on *S. rugosoannulata* cultivation, soil microbial ecology, and disease suppression through field experiments. Substitution of 10% tobacco stalk in corn straw substrates achieved a synergistic effect of high yield and excellent commercial quality in *S. rugosoannulata*. The TC group further optimized community function and significantly strengthened the disease suppression potential driven by microorganisms. AP, AK, and pH emerged as primary drivers of microbial community differentiation. Our findings present an innovative strategy integrating ecological restoration with high-value biomass production for sustainable straw resource utilization. Nevertheless, long-term positioning experiments and mechanism analyses (e.g., metagenomics and metabolomics) are still needed to deepen the understanding of the regulatory network.

## Data Availability

All sequence data have been deposited in NCBI Sequence Read Archive database under accession number PRJNA1307271.
